# Interfacial Spin–Orbit-Coupling-Induced Strong Spin-to-Charge Conversion at an All-Oxide Ferromagnetic/Quasi-Two-Dimensional Electron Gas Interface

**DOI:** 10.1021/acsami.4c20213

**Published:** 2025-03-13

**Authors:** Mi-Jin Jin, Guang Yang, Doo-Seung Um, Jacob Linder, Jason W.A. Robinson

**Affiliations:** 1 Center for Multidimensional Carbon Materials (CMCM), Institute for Basic Science (IBS), Ulsan 44919, Republic of Korea; 2 Department of Materials Science & Metallurgy, University of Cambridge, 27 Charles Babbage Road, Cambridge CB3 0FS, United Kingdom; 3 School of Integrated Circuit Science and Engineering, Beihang University, Beijing 100191, China; 4 Department of Electronic Engineering, Jeju National University (JNU), Jeju-do63243, Korea; 5 Center for Quantum Spintronics, Department of Physics, Norwegian University of Science and Technology, Trondheim NO-7491, Norway

**Keywords:** spin−orbit coupling, spin Hall effect, spin−charge conversion, oxide interface, ferromagnetic resonance

## Abstract

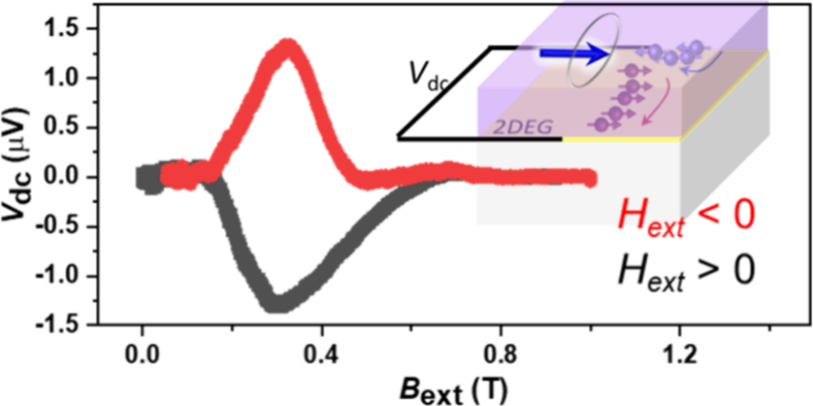

Functional oxides and hybrid structures with interfacial spin–orbit coupling and the Rashba–Edelstein effect (REE) are promising materials systems for thermal tolerance spintronic device applications. Here, we demonstrate efficient spin-to-charge conversion through enhanced interfacial spin–orbit coupling at the all-oxide interface of La_1–*x*_Ca_*x*_MnO_3_ with quasi-two-dimensional (quasi-2D) SrTiO_3_ (LCMO/STO). The quasi-2D interface is generated via oxygen vacancies at the STO surface. We obtain a spin-to-charge conversion efficiency of θ_∥_ ≈ 2.32 ± 1.3 nm, most likely originating from the inverse REE, which is relatively large versus all-metallic spin-to-charge conversion materials systems. The results highlight that the LCMO/STO 2D electron gas is a potential platform for spin-based memory and transistor applications.

## Introduction

In spintronics, the spin Hall effect (SHE) originates from spin–orbit coupling (SOC), which plays an important role in spin-to-charge interconversion, magnetization switching, and spin current manipulation.^1−20^ Nevertheless, the SHE and inverse SHE are bulk phenomena with three-dimensional propagation of spin. Recent reports have shown that the interfacial SOC effect from the Rashba–Edelstein effect (REE), the inverse Rashba–Edelstein effect (IREE), or the spin Galvanic effect (SGE) is key for spin and/or charge generation and detection in low dimensional systems.^1,21−28^ Rashba SOC arises due to spatial broken symmetry at surfaces and interfaces, lifting spin degeneracy and causing locking between momentum and spin degrees of freedom. At symmetry broken interfaces, charge flow creates a nonzero spin accumulation (i.e., the REE).

SrTiO_3_ (STO) has a highly tunable quasi-2D conductivity,^29^ intrinsic/extrinsic SOC,^30^ structural symmetry broken intrinsic Rashba interface,^31^ and ferroelectricity.^22^ STO is, therefore, promising for artificially manipulating interface properties with oxide ferromagnetic layers—see, e.g., studies on oxide magnetic thin films including La_*x*_Ca_1–*x*_MO_3_ (LCMO), La_*x*_Sr_1–*x*_MnO_3_ (LSMO), and yttrium iron garnet (YIG).^32−42^ However, studies on the interaction between such a magnetic oxide layer and a conductive oxide surface layer remain challenging and topical, since they are important for the development of spin–orbitronic and spin–interface electronics applications.

Ferromagnetic resonance (FMR) spin pumping is an established technique for probing magnetic dynamic properties of ferromagnetic materials, including spin-to-charge conversion (or vice versa). Recently, spin currents generated by an REE-driven spin accumulation has been reported using FMR with spin pumping in CoFeB/LaAlO_3_/SrTiO_3_ structures,^43^ and spin generated by charge on interfacial conducting layers in NiFe/Al/SrTiO_3_.^44^ Charge generated by spin on ferromagnetic layers via FMR with spin pumping has also been reported in Py/LaAlO_3_/SrTiO_3_,^45^ NiFe/Al/SrTiO_3_,^21,22^ and La_0.67_Sr_0.33_MnO_3_/LaAlO_3_/SrTiO_3_^31^ heterostructures, and at NiFe/Bi_2_Se_3_ and Y_3_Fe_5_O_12_/Bi_2_Se_3_,^25^ Pt/NiFe_2_, and graphene/YIG^46^ interfaces.

Here, we report efficient conversion of spin currents into charge currents via the FMR of LCMO on STO and simultaneous spin pumping at the quasi-2D STO interface. The spin-to-charge conversion efficiency caused by IREE is θ_∥_ ≈ 2.32 ± 1.3 nm at 5 K, which is relatively large versus all-metal-based materials systems. Our results can lead to potential applications in oxide-based systems for low-power detection and generation of spin in nonmagnetic systems.

## Results and Discussion

### Basic Properties of LCMO/Quasi-2D STO

**Figure 1:**
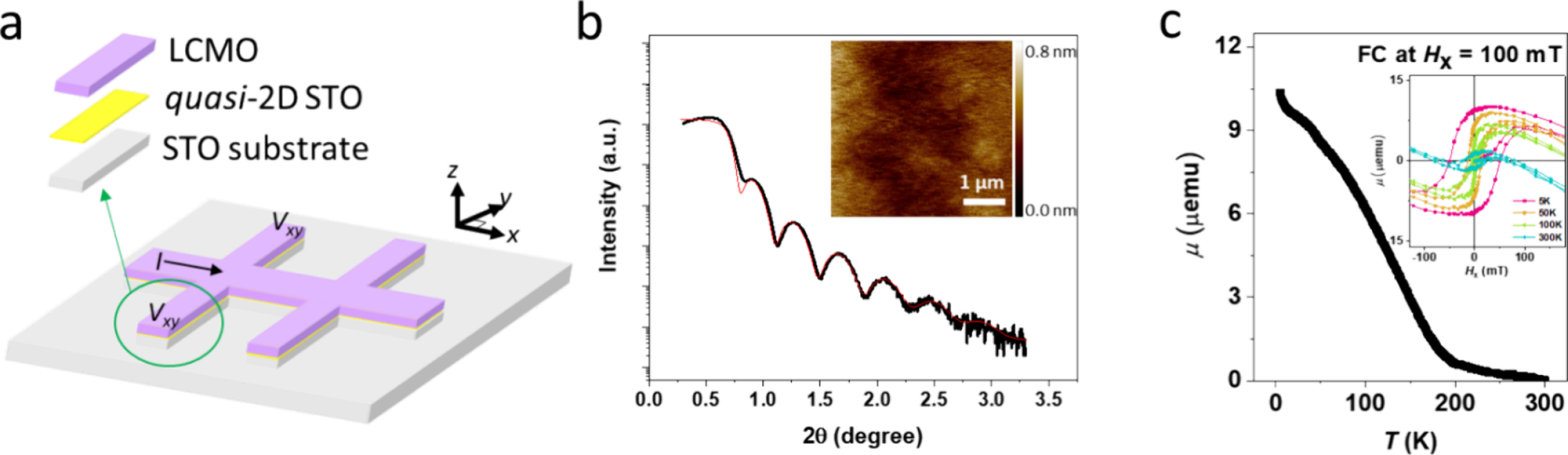
(a) Schematic diagram of the (001)-oriented STO LCMO/STO Hall bar. (b) X-ray reflectivity (XRR) of the LCMO/STO stack (red line is the fitted data). 2θ is the angle between the transmitted X-ray beam and the reflected X-ray beam. The inset image shows the surface morphology of LCMO measured by atomic force microscopy (AFM). (c) Magnetic properties measured using a vibrating sample magnetometer. Magnetic moment (μ) vs temperature (*T*) at 100 mT (field cooling, FC) and magnetic moment (μ) vs field sweep (*H*_*z*_) at different temperatures [5, 50, 100, and 300 K] (inset).

A conductive quasi-2D interface was artificially created between (001)-oriented STO and LCMO using Ar plasma treatment. As shown in Figure 1a, the sample was fabricated with a six-armed Hall bar device (500 μm channel width, 1500 μm channel length) using optical lithography, reactive ion etching, and thermal annealing.^29^ The LCMO has a thickness of 15 nm, determined by X-ray reflectivity (XRR) (Figure 1b) using GenX fitting software. We used La_0.7_Ca_0.3_MnO_3_ source material for pulsed laser deposition (PLD). The X-ray diffraction (XRD) was used to analyze and optimize LCMO on STO (100) film growth (Supporting Information Figure S1). Atomic force microscopy (AFM) on the quasi- 2D surface (Figure 1b inset) shows that the LCMO surface is smooth with a vertical root-mean-square roughness of 1 nm over a 5 × 5 μm^–2^ area. For more information, we added two different sample surfaces highlighting how growth of LCMO depends on the state quality of STO (see Figure S2 in the Supporting Information). Volumetric magnetic properties of the LCMO are investigated using a vibrating sample magnetometer (VSM) at different temperatures and magnetic fields. From the VSM measurements, can estimate a magnetically dead layer in LCMO of about 5 nm (Figure S4). In Figure 1c, we plotted the temperature dependence of the magnetic moment (field cooling, FC) with an external magnetic field of 100 mT after Hall bar geometry device fabrication. The LCMO shows a rise in magnetic moment with decreasing temperature, reaching a maximum moment of 10 μemu at 2 K, from which we estimate a Curie temperature (*T*_c_) of 175 K. Note that the LCMO magnetic properties and Curie temperature are thickness dependent.^47^ The inset in Figure 1c shows magnetic moment vs in-plane magnetic field hysteresis loops for the LCMO Hall bar at four different temperatures. At 2 K, the magnetic coercivity of the LCMO is about 100 mT.

**Figure 2:**
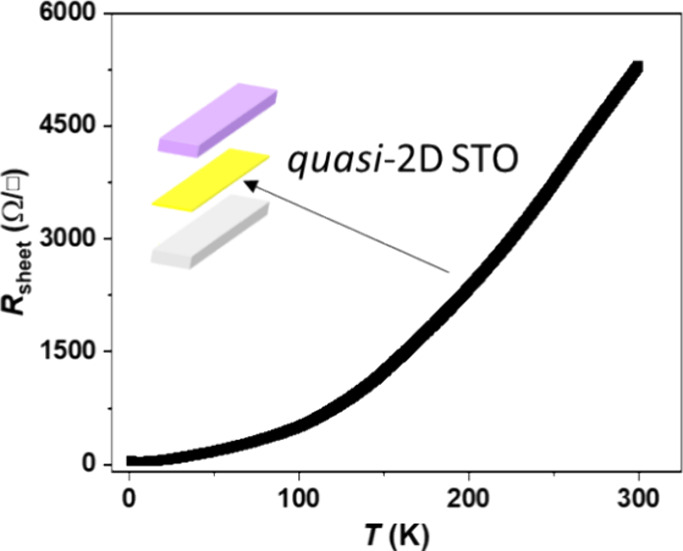
Sheet resistance (*R*_S_) vs temperature (*T*) of the quasi-2D STO interface. *I*_source_ = 1 μA. The STO conducting interface shows metallic behavior over the temperature range from 2 to 300 K.

Next, we investigate the quasi-2D interface of STO (after post deposition of LCMO and Hall bar pattern). The temperature dependence of the sheet resistance is shown in Figure 2, confirming that the quasi-2D interface of STO is stable with metallic behavior and a sheet resistance of ∼50 Ω/□ at 2 K. Note that the LCMO layer shows a semiconductor-like behavior and a different resistance range. Supporting Information Figure S3 shows the temperature-dependent sheet resistance of LCMO. Compared with Figure 2, the behavior in Figure S3 demonstrates that the quasi-2D conducting interface is stable with a metallic behavior through the temperature range. From the Hall effect and sheet resistance, we estimated a carrier concentration of the quasi-2D interface of *n*_s_ = BI/(*q*|*V*_H_|) = 1/(*q*μ_s_*R*_s_) ≈ 1.63 × 10^13^ cm^–2^ and mobility μ_s_ = |*V*_H_|/(BIR_s_) = 1/(*qn*_s_*R*_s_) ≈ 11,715 cm^2^/V·s at 5 K. Here, *q* is the electron charge (1.602 × 10^–19^ C).

### FMR with Spin Pumping_Spin-to-Charge Conversion

**Figure 3:**
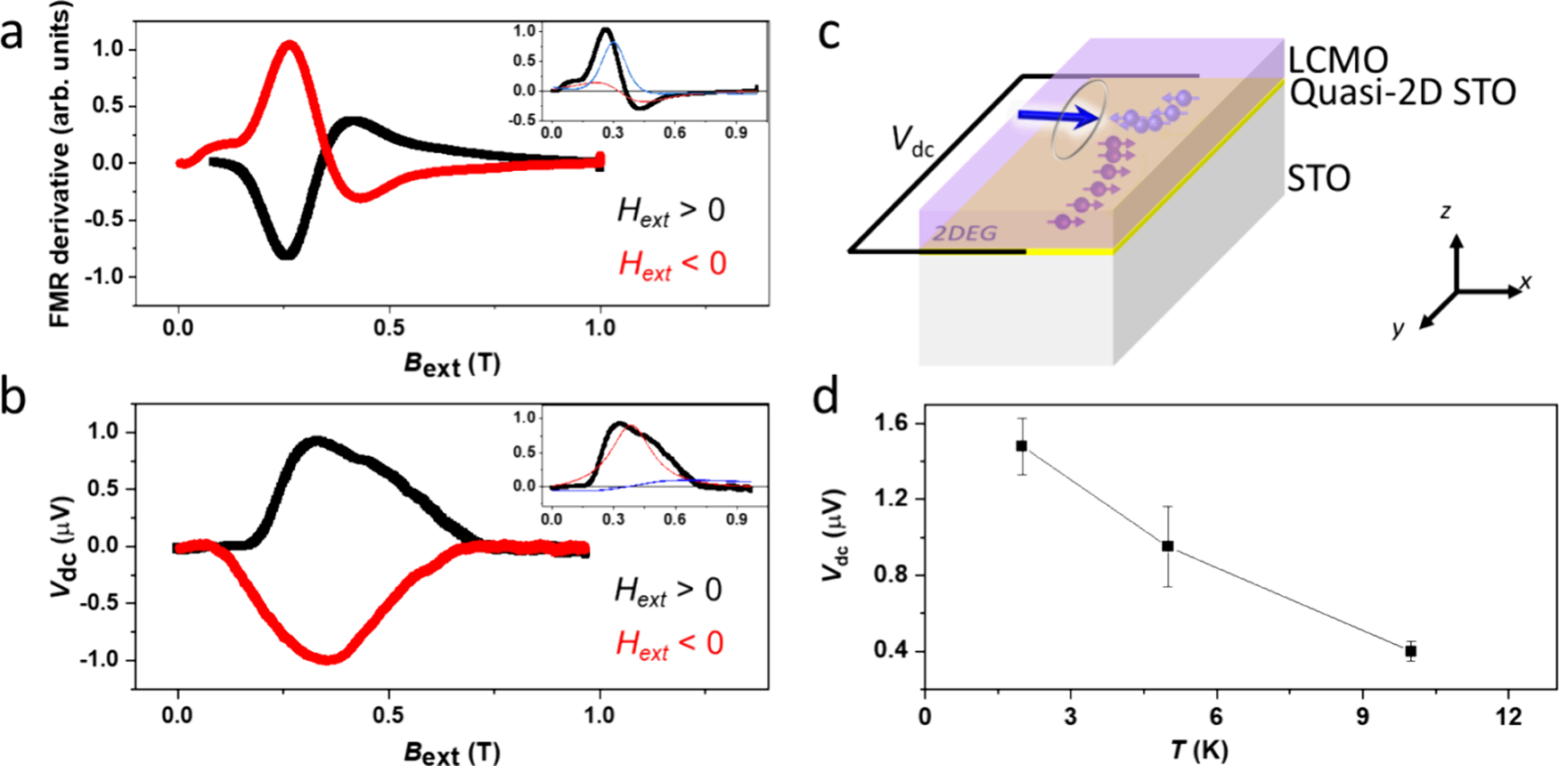
(a) FMR signal from LCMO on the (001)-oriented STO. (Inset) FMR signal fitted with a symmetry component (blue) and an asymmetry component (red). (b) Spin pumping signal detected via FMR. Spin-based pumping voltage signal detected from the conducting quasi-2D STO layer at positive and negative external fields. (Inset) Spin-based pumping signal fitted with a symmetry component (blue) and an asymmetry component (red). The FMR peak and spin pumping measurement were taken at 5 K. (c) Schematic illustration with spin pumping measurement LCMO/quasi-2D STO/STO. (d) Temperature vs spin pumping voltage *V*_s,dc_ where the solid lines are a guide to the eye. The spin-to-charge conversion voltage gradually decreases with increasing temperature.

FMR spin pumping measurements are used for evaluating interfacial SOC^21,43,45^ at a ferromagnetic/conducting layer interface. Figure 3c shows a schematic diagram of the FMR setup with spin pumping on the LCMO/quasi-2D STO/STO structure. Figure 3a shows a representative FMR signal from LCMO/STO at a microwave frequency of 8 GHz and a power of 25 dBm. The LCMO shows a typical FMR response (Lamor precession) as the microwave voltage passes along the LCMO surface (Figure 3a). Simultaneously, a spin current from LCMO is injected into the interface, generating a nonequilibrium spin distribution. The resulting electron spin distribution leads to a voltage difference *V*_dc_ across the direction parallel to the interface (Figure 3b). The generated voltage by spin pumping likely arises due to the strong SOC at the LCMO/STO interface. Therefore, the spin pumping voltage (i.e., *V*_dc_) is evidence of strong SOC at the quasi-2D interface. The FMR derivative can be fitted using *V*_FMR derivative_ = *V*_symmetry_*F*_symmetry_ + *V*_Asymmetry_*F*_Asymmetry_, where *F*_s_ and *F*_A_ are the symmetric and antisymmetric Lorentzian functions, respectively. As shown in the inset of Figure 3a, the amplitude of the symmetric voltage (*V*_symmetry_) and the antisymmetric voltage (*V*_Asymmetry_) in the inset of Figure 3a (red and blue fitted lines) is correlated and is related to damping-like- and field-like-torque terms, respectively.^43,48^ The relatively large symmetric component of *V*_s_*F*_s_ suggests an in-plane spin polarization, but not exact, damping-like torque is dominant. Also, the spin pumping signal (*V*_dc_) can be analyzed for the symmetric and antisymmetric components, correlating SOC strength with spin pumping and anisotropic magnetoresistance, and a planar Hall effect,^23,49−51^ respectively. A large symmetric component of *V*_s,dc_*F*_s,dc_ as shown in Figure 3b inset (red fitted line) suggests that a large SOC-related spin pumping is dominant, even though an antisymmetric component still exists [Figure 3b inset (blue fitted line)].^2^ We separately plot the spin-to-charge conversion voltage *V*_s,dc_ as a function of temperatures in Figure 3d. The spin-to-charge conversion voltage diminishes with increasing temperature. The original plots of LCMO FMR with the spin pumping signal at 2 K (Figure S5a,b) and at 10 K (Figure S5c,d) are shown in the Supporting Information, Part 4.

**Figure 4:**
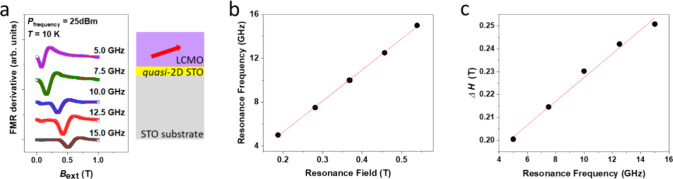
(a) FMR signal from LCMO vs DC magnetic field with different applied microwave frequencies (labeled). Right illustration shows the cross-sectional structure of the device with the (red) arrow indicating the likely magnetic anisotropy direction of the LCMO with respect to interface. (b) Resonance frequency (GHz) vs resonance field, fitted using the Kittel’s relation (red line). (c) Peak-to-peak line width Δ*H* depends on resonance frequency (GHz). From fitting of (b) and (c) plots, we determine the characteristic values of the effective magnetization and Gilbert damping constant of the LCMO layer. Solid (red) lines are fits, as explained in the main text.

To investigate further the magnetic properties of the LCMO layer, we measured the FMR signal versus FMR frequency. Figure 4a shows the dynamic magnetic resonance at several different frequencies *f* of the microwave excitation, versus external magnetic field *H*_ext_. The corresponding frequency dependence of the resonance field and the first derivative of the resonance peak-to-peak line width Δ*H* are reported in Figure 4b and Figure 4c, respectively. The dispersion relation of *H*_res_ with *f* follows the Kittel relation (31,52,53) where γ is the gyromagnetic ratio, *H*_res_is the resonance magnetic field, and *M*_eff_ is the effective magnetization (0.02 ± 0.01) kA/m. The effective Gilbert damping constant α_LCMO/quasi-2D STO_is determined by fitting the frequency dependence of the peak-to-peak line width Δ*H* (Figure 4c) using , where Δ*H*_0_ is the frequency-independent contribution from magnetic inhomogeneity in LCMO. The Gilbert damping α_LCMO/quasi-2D STO_ is estimated to be about 0.19 ± 0.1 at 10 K.

The spin-to-charge conversion efficiency is related to SOC via the relation θ_∥_ = *j*_c_^2D^/*j*_s_. A quantitative analysis of spin-to-charge conversion requires the pumped spin current density to be determined using the relationship as follows:^21,31,52,54^
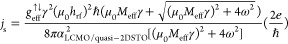
1where *g*_eff_^↑↓^ is the effective spin mixing conductance (,^21^,^31^) γ is the gyromagnetic ratio, *h*_rf_ is the microwave field amplitude, ω = 2π*f*, and *t*_FM_ = 15 nm is the thickness of the LCMO film. From the above, we obtain average *g*_eff_^↑↓^ ≈ 7.03 × 10^16^ m^–2^, which is reasonable versus values reported elsewhere at interfacial systems.^55^*j*_s_ ∼ 2.5 × 10^4^ A m^–2^, which, in conjunction with the measured 2D charge current, results in a spin-to-charge current conversion efficiency of θ_∥_ = *j*_c_^2D^/*j*_s_ ≈ 2.32 ± 1.3 nm. This value in our LCMO/quasi-2D STO/STO structure is relatively large versus metal based interfaces^53,55−57^ but also consistent with other oxide systems.^21−23,31^

## Discussion

The spin-to-charge conversion at the quasi-2D interface most likely originates from IREE. An injected spin accumulation will generate a charge current flowing parallel to the interface, causing a measurable voltage across the ends of the sample. For the IREE case, the band structure is split due to the Rashba effect at the oxide interface. The spin current from the LCMO is injected into the LCMO/quasi-2D STO interface at FMR, generating a charge current in the lateral (*y*) direction (see Figure 3c). This effect induces an electromotive force between the electrodes at the edges of the sample along the long *y*. The electromotive force includes a signal from the LCMO that is induced by the microwave electric field through the galvanomagnetic effects (ex. anomalous Hall effect and planar Hall effect), which should be separated from the IREE signal, as shown in Figure 3. The possible origin of this IREE could relate to interfacial stemming from the broken inversion symmetry at the interface, or impurity (such as oxygen vacancy)-induced extrinsic interfacial spin orbit interaction.^58−60^ Note that the sign of the spin pumping can sometimes be the same value for opposite external magnetic fields. This behavior may have two possible origins: one, if the ferroelectric polarity of STO is unstable;^22^ two, a competition between spin and orbital Edelstein effects.^61^

## Conclusions

In this study, we have experimentally demonstrated a strong spin current to charge current conversion at an all-oxide interface between LCMO and STO using FMR spin pumping. In particular, we show strong spin-to-charge conversion via SOC at the quasi-2D LCMO/STO interface. From these results, we obtain a spin-to-charge conversion efficiency of θ_∥_ ≈ 2.32 ± 1.3 nm, which originates from interfacial SOC through the spin diffusion length. The value range is relatively large versus metallic-based systems. Our results highlight that two-dimensional electron gases with functional oxide layers are strong candidates for spintronics and spin–orbitronic low-power devices.

## Methods

### Sample Preparation

5 mm × 5 mm single crystal (100)-oriented (with <0.1° tolerance from CrysTec GmbH) STO substrates were used. Substrates were cleaned by acetone, ethanol, and deionized water with sonication. Ar plasma treatment was followed to creating oxygen vacancies on the STO surface, which leads to a conductive surface.^29^ In-situ Ar plasma (50 W power) treatment was applied to the STO surface for 30 min at a pressure of 30 mTorr (base pressure up to 10^–9^ Torr). After that, the sample was directly transferred to a PLD system chamber and allowed to stand until the base pressure was in the 10^–6^ Torr range. Finally, a magnetic oxide layer of La_1–*x*_Ca_*x*_MnO_3_ (LCMO) was deposited onto STO. We used La_0.7_Ca_0.3_MnO_3_ as the target material. For the LCMO deposition, a laser pulse of 3 Hz with an energy density ≈2 J cm^–2^ was applied at 700 °C with oxygen pressure 1 × 10^–1^ Torr. This condition gives an approximate growth rate of 1 nm per minute.

### Device Fabrications

The simple Hall bar-shaped geometry is chosen for not only applying longitudinal charge current but also generating an SHE-induced transverse voltage detection. For the patterning, a simply patterned shadow mask (channel width 500 μm and channel length 1500 μm) is used for the buffer layer deposition. A 100 nm-thick Al layer is then deposited as a buffer layer. After then, controlled (working pressure 20 mTorr) oxygen plasma treatment (Adixen, AMS 100) followed by oxygen annealing (3 h, 300 °C) is used to form the Hall bar patterned conductive STO.^29,62^ Finally, the Al buffer layer is removed by using a base solution. Detailed fabrication processes are described in the previous work.^29^ After patterning, Au (30 nm)/Ti (10 nm) layers are deposited (thermal and e-beam evaporation) for contact electrodes and direct conducting STO interface contact.

### Low-Temperature Analysis Measurements

Siver paste or indium paste was used to connect between sample electrodes and the measurement equipment puck through copper wires. Temperature-dependent sheet resistance and low-temperature magnetic field sweep-dependent signal detection related with magnetization switching were studied in liquid helium dewar system with a source meter (Keithley 6221, Keithley 2636) and a nanovoltmeter (Keithley 2182). Spin pumping followed by ferromagnetic resonance (FMR) measurement was studied using the Physical Property Measurement System (Quantum Design) with a lock-in amplifier (SR830), an AC source meter (Keithley 6221), and a nanovoltmeter (Keithley 2182).

## Supporting Information Available

X-ray diffraction analysis; AFM image of sample surface; LCMO thin film’s temperature-dependent sheet resistance; dead layer estimation; and FMR and spin pumping data at 2 and 10 K (PDF)

## References

[ref1] Frangou L.; Oyarzun S.; Auffret S.; Vila L.; Gambarelli S.; Baltz V. Enhanced Spin Pumping Efficiency in Antiferromagnetic IrMn Thin Films around the Magnetic Phase Transition. Phys. Rev. Lett. 2016, 116, 077203 10.1103/PhysRevLett.116.077203. 26943556

[ref2] Jeon K. R.; Montiel X.; Komori S.; Ciccarelli C.; Haigh J.; Kurebayashi H.; Cohen L. F.; Chan A. K.; Stenning K. D.; Lee C. M.; Eschrig M.; Blamire M. G.; Robinson J. W. A. Tunable Pure Spin Supercurrents and the Demonstration of Their Gateability in a Spin-Wave Device. Phys. Rev. X 2020, 10, 031020 10.1103/PhysRevX.10.031020.

[ref3] Hibino Y.; Taniguchi T.; Yakushiji K.; Fukushima A.; Kubota H.; Yuasa S. Giant charge-to-spin conversion in ferromagnet via spin-orbit coupling. Nat. Commun. 2021, 12, 6254 10.1038/s41467-021-26445-y. 34716327 PMC8556288

[ref4] Roy K.; Nayak S.; Gupta P.; Bedanta S. Spin dynamics and inverse spin Hall effect study in the metallic Pt/NiMn/CoFeB system. Phys. Chem. Chem. Phys. 2022, 24, 24323–24327. 10.1039/D2CP01857H. 36173608

[ref5] Shinozaki M.; Igarashi J.; Iwakiri S.; Kitada T.; Hayakawa K.; Jinnai B.; Otsuka T.; Fukami S.; Kobayashi K.; Ohno H. Nonlinear conductance in nanoscale CoFeB/MgO magnetic tunnel junctions with perpendicular easy axis. Phys. Rev. B 2023, 107, 094436 10.1103/PhysRevB.107.094436.

[ref6] Saito Y.; Ikeda S.; Tezuka N.; Inoue H.; Endoh T. Field-free spin-orbit torque switching and large dampinglike spin-orbit torque efficiency in synthetic antiferromagnetic systems using interfacial Dzyaloshinskii-Moriya interaction. Phys. Rev. B 2023, 108, 024419 10.1103/PhysRevB.108.024419.

[ref7] Zhou T.; Zhou S.; Xie X.; Zhao X.; Dong Y.; Wang J.; Chen W.; Leng Q.; Bai L.; Chen Y.; Kang S.; Liu Y.; Yan S.; Tian Y. Perpendicular effective field induced by spin-orbit torque and magnetization damping in chiral domain walls. Phys. Rev. B 2023, 107, 104411 10.1103/PhysRevB.107.104411.

[ref8] Figueiredo-Prestes N.; Tsipas P.; Krishnia S.; Pappas P.; Peiro J.; Fragkos S.; Zatko V.; Lintzeris A.; Dlubak B.; Chaitoglou S.; Heuken M.; Reyren N.; Jaffrès H.; Seneor P.; Dimoulas A.; George J. M. Large Fieldlike Spin-Orbit Torque and Magnetization Manipulation in a Fully Epitaxial van der Waals Two-Dimensional-Ferromagnet/Topological-Insulator Heterostructure Grown by Molecular-Beam Epitaxy. Phys. Rev. Appl. 2023, 19, 014012 10.1103/PhysRevApplied.19.014012.

[ref9] Liu R.; Zhang Y.; Yuan Y.; Lu Y.; Liu T.; Chen J.; Wei L.; Wu D.; You B.; Zhang W.; Du J. Manipulating exchange bias in Ir25Mn75/CoTb bilayer through spin–orbit torque. Appl. Phys. Lett. 2023, 122, 062401 10.1063/5.0139997.

[ref10] Fan H.; Jin M.; Luo Y.; Yang H.; Wu B.; Feng Z.; Zhuang Y.; Shao Z.; Yu C.; Li H.; Wen J.; Wang N.; Liu B.; Li W.; Zhou T. Field-Free Spin-Orbit Torque Switching in Synthetic Ferro and Antiferromagents with Exchange Field Gradient. Adv. Funct. Mater. 2023, 33, 2211953 10.1002/adfm.202211953.

[ref11] Yamamoto T.; Nozaki T.; Imamura H.; Tamaru S.; Yakushiji K.; Kubota H.; Fukushima A.; Yuasa S. Voltage-Driven Magnetization Switching Controlled by Microwave Electric Field Pumping. Nano Lett. 2020, 20, 6012–6017. 10.1021/acs.nanolett.0c02022. 32649831

[ref12] Krishnia S.; Sassi Y.; Ajejas F.; Sebe N.; Reyren N.; Collin S.; Denneulin T.; Kovacs A.; Dunin-Borkowski R. E.; Fert A.; George J. M.; Cros V.; Jaffres H. Large Interfacial Rashba Interaction Generating Strong Spin-Orbit Torques in Atomically Thin Metallic Heterostructures. Nano Lett. 2023, 23, 6785–6791. 10.1021/acs.nanolett.2c05091. 37524333 PMC10416352

[ref13] Zhu D.; Zhang T.; Fu X.; Hao R.; Hamzic A.; Yang H.; Zhang X.; Zhang H.; Du A.; Xiong D.; Shi K.; Yan S.; Zhang S.; Fert A.; Zhao W. Sign Change of Spin-Orbit Torque in Pt/NiO/CoFeB Structures. Phys. Rev. Lett. 2022, 128, 217702 10.1103/PhysRevLett.128.217702. 35687442

[ref14] Kao I. H.; Muzzio R.; Zhang H.; Zhu M.; Gobbo J.; Yuan S.; Weber D.; Rao R.; Li J.; Edgar J. H.; Goldberger J. E.; Yan J.; Mandrus D. G.; Hwang J.; Cheng R.; Katoch J.; Singh S. Deterministic switching of a perpendicularly polarized magnet using unconventional spin-orbit torques in WTe(2). Nat. Mater. 2022, 21, 1029–1034. 10.1038/s41563-022-01275-5. 35710631

[ref15] Ryu J.; Thompson R.; Park J. Y.; Kim S.-J.; Choi G.; Kang J.; Jeong H. B.; Kohda M.; Yuk J. M.; Nitta J.; Lee K.-J.; Park B.-G. Efficient spin–orbit torque in magnetic trilayers using all three polarizations of a spin current. Nat. Elec. 2022, 5, 217–223. 10.1038/s41928-022-00735-9.

[ref16] Liu L.; Qin Q.; Lin W.; Li C.; Xie Q.; He S.; Shu X.; Zhou C.; Lim Z.; Yu J.; Lu W.; Li M.; Yan X.; Pennycook S. J.; Chen J. Current-induced magnetization switching in all-oxide heterostructures. Nat. Nanotechnol. 2019, 14, 939–944. 10.1038/s41565-019-0534-7. 31501531

[ref17] Zhang Q.; Zhao Y.; He C.; Huo Y.; Cui B.; Zhu Z.; Zhang G.; Yu G.; He B.; Zhang Y.; Lyu H.; Guo Y.; Qi J.; Shen S.; Wei H.; Shen B.; Wang S. Perpendicular Magnetization Switching Driven by Spin-Orbit Torque for Artificial Synapses in Epitaxial Pt-Based Multilayers. Adv. Elec. Mater. 2022, 8, 2200845 10.1002/aelm.202200845.

[ref18] Oh I.; Park J.; Choe D.; Jo J.; Jeong H.; Jin M. J.; Jo Y.; Suh J.; Min B. C.; Yoo J. W. A scalable molecule-based magnetic thin film for spin-thermoelectric energy conversion. Nat. Commun. 2021, 12, 1057 10.1038/s41467-021-21058-x. 33594084 PMC7887260

[ref19] Liu L.; Pai C. F.; Li Y.; Tseng H. W.; Ralph D. C.; Buhrman R. A. Spin-Torque Switching with the Giant spin hall effect. Science 2012, 336, 555–558. 10.1126/science.1218197. 22556245

[ref20] Miron I. M.; Garello K.; Gaudin G.; Zermatten P. J.; Costache M. V.; Auffret S.; Bandiera S.; Rodmacq B.; Schuhl A.; Gambardella P. Perpendicular switching of a single ferromagnetic layer induced by in-plane current injection. Nature 2011, 476, 189–193. 10.1038/nature10309. 21804568

[ref21] Lesne E.; Fu Y.; Oyarzun S.; Rojas-Sanchez J. C.; Vaz D. C.; Naganuma H.; Sicoli G.; Attane J. P.; Jamet M.; Jacquet E.; George J. M.; Barthelemy A.; Jaffres H.; Fert A.; Bibes M.; Vila L. Highly efficient and tunable spin-to-charge conversion through Rashba coupling at oxide interfaces. Nat. Mater. 2016, 15, 1261–1266. 10.1038/nmat4726. 27571452

[ref22] Noel P.; Trier F.; Vicente Arche L. M.; Brehin J.; Vaz D. C.; Garcia V.; Fusil S.; Barthelemy A.; Vila L.; Bibes M.; Attane J. P. Non-volatile electric control of spin-charge conversion in a SrTiO(3) Rashba system. Nature 2020, 580, 483–486. 10.1038/s41586-020-2197-9. 32322081

[ref23] Vaz D. C.; Noel P.; Johansson A.; Gobel B.; Bruno F. Y.; Singh G.; McKeown-Walker S.; Trier F.; Vicente-Arche L. M.; Sander A.; Valencia S.; Bruneel P.; Vivek M.; Gabay M.; Bergeal N.; Baumberger F.; Okuno H.; Barthelemy A.; Fert A.; Vila L.; Mertig I.; Attane J. P.; Bibes M. Mapping spin-charge conversion to the band structure in a topological oxide two-dimensional electron gas. Nat. Mater. 2019, 18, 1187–1193. 10.1038/s41563-019-0467-4. 31501554

[ref24] Mello K.; Abrão J. E.; Santos E. S.; Mendes J. B. S.; Raposo E. P.; Azevedo A. Unraveling the spin current flow in Bi layers. Phys. Rev. B 2022, 106, 214418 10.1103/PhysRevB.106.214418.

[ref25] Mendes J. B. S.; Gamino M.; Cunha R. O.; Abrão J. E.; Rezende S. M.; Azevedo A. Unveiling the spin-to-charge current conversion signal in the topological insulator Bi2Se3 by means of spin pumping experiments. Phys. Rev. Mater. 2021, 5, 024206 10.1103/PhysRevMaterials.5.024206.

[ref26] Hao R.; Zhang K.; Chen W.; Qu J.; Kang S.; Zhang X.; Zhu D.; Zhao W. Significant Role of Interfacial Spin-Orbit Coupling in the Spin-to-Charge Conversion in Pt/NiFe Heterostructure. ACS Appl. Mater. Interfaces 2022, 14, 57321–57327. 10.1021/acsami.2c13434. 36525266

[ref27] Lee A. J.; Ahmed A. S.; McCullian B. A.; Guo S.; Zhu M.; Yu S.; Woodward P. M.; Hwang J.; Hammel P. C.; Yang F. Interfacial Rashba-Effect-Induced Anisotropy in Nonmagnetic-Material-Ferrimagnetic-Insulator Bilayers. Phys. Rev. Lett. 2020, 124, 257202 10.1103/PhysRevLett.124.257202. 32639765

[ref28] Fan Y.; Upadhyaya P.; Kou X.; Lang M.; Takei S.; Wang Z.; Tang J.; He L.; Chang L. T.; Montazeri M.; Yu G.; Jiang W.; Nie T.; Schwartz R. N.; Tserkovnyak Y.; Wang K. L. Magnetization switching through giant spin-orbit torque in a magnetically doped topological insulator heterostructure. Nat. Mater. 2014, 13, 699–704. 10.1038/nmat3973. 24776536

[ref29] Jin M.-J.; Choe D.; Lee S. Y.; Park J.; Jo J.; Oh I.; Kim S.-I.; Baek S.-H.; Jeon C.; Yoo J.-W. Probing surface electronic properties of a patterned conductive STO by reactive ion etching. Appl. Surf. Sci. 2019, 466, 730–736. 10.1016/j.apsusc.2018.10.068.

[ref30] Jin M. J.; Um D. S.; Ohnishi K.; Komori S.; Stelmashenko N.; Choe D.; Yoo J. W.; Robinson J. W. A. Pure Spin Currents Driven by Colossal Spin-Orbit Coupling on Two-Dimensional Surface Conducting SrTiO(3). Nano Lett. 2021, 21, 6511–6517. 10.1021/acs.nanolett.1c01607. 34320314

[ref31] Ohya S.; Araki D.; Anh L. D.; Kaneta S.; Seki M.; Tabata H.; Tanaka M. Efficient intrinsic spin-to-charge current conversion in an all-epitaxial single-crystal perovskite-oxide heterostructure of La0.67Sr0.33MnO3/LaAlO3/SrTiO3. Phys. Rev. Res. 2020, 2, 012014 10.1103/PhysRevResearch.2.012014.

[ref32] Haspot V.; Noël P.; Attané J.-P.; Vila L.; Bibes M.; Anane A.; Barthélémy A. Temperature dependence of the Gilbert damping of La0.7Sr0.3MnO3 thin films. Phys. Rev. Mater. 2022, 6, 024406 10.1103/PhysRevMaterials.6.024406.

[ref33] Sinha U. K.; Sahoo A.; Padhan P. Enhanced low-field positive magnetoresistance and magnetic anisotropy in La0.7Sr0.3MnO3 films grown on (001) Si. J. Alloys Compd. 2023, 952, 170037 10.1016/j.jallcom.2023.170037.

[ref34] Fang F.; Yin Y. W.; Li Q.; Lüpke G. Spin-polarized current injection induced magnetic reconstruction at oxide interface. Sci. Rep. 2017, 7, 40048 10.1038/srep40048. 28051142 PMC5209677

[ref35] Guo J.; He B.; Han Y.; Liu H.; Han J.; Ma X.; Wang J.; Gao W.; Lu W. Resurrected and Tunable Conductivity and Ferromagnetism in the Secondary Growth La(0.7)Ca(0.3)MnO(3) on Transferred SrTiO(3) Membranes. Nano Lett. 2024, 24, 1114–1121. 10.1021/acs.nanolett.3c03651. 38252877

[ref36] Mikailzade F.; Önal F.; Maksutoglu M.; Zarbali M.; Göktaş A. Structure and Magnetization of Polycrystalline La0.66Ca0.33MnO3 and La0.66Ba0.33MnO3 Films Prepared Using Sol-Gel Technique. J. Supercond. Nov. Magn. 2018, 31, 4141–4145. 10.1007/s10948-018-4683-y.

[ref37] NHUNG N. T. M.; KIM H. J. Study on the Structural and Electrical Properties of La0.7Ca0.3MnO3/CaMnO3/La0.7Ca0.3MnO3 Heterostructures. New Phys.: Sae Mulli 2021, 71, 446–449. 10.3938/NPSM.71.446.

[ref38] Zhao B.; Hu X.; Dong F.; Wang Y.; Wang H.; Tan W.; Huo D. The Magnetic Properties and Magnetocaloric Effect of Pr0.7Sr0.3MnO3 Thin Film Grown on SrTiO3 substrate. Mater. 2023, 16, 75 10.3390/ma16010075. PMC9821194 36614413

[ref39] Safarina G. A.; Kim Y. J.; Park H. S.; Yang C. H. Raman spectroscopy of the Jahn–Teller phonons in a magnetic LaMnO3 thin film grown on KTaO3. J. Appl. Phys. 2022, 131, 025302 10.1063/5.0076160.

[ref40] Yang M.; Sun L.; Zeng Y.; Cheng J.; He K.; Yang X.; Wang Z.; Yu L.; Niu H.; Ji T.; Chen G.; Miao B.; Wang X.; Ding H. Highly efficient field-free switching of perpendicular yttrium iron garnet with collinear spin current. Nat. Commun. 2024, 15, 3201 10.1038/s41467-024-47577-x. 38615046 PMC11016059

[ref41] Wei X. Y.; Santos O. A.; Lusero C. H. S.; Bauer G. E. W.; Ben Youssef J.; van Wees B. J. Giant magnon spin conductivity in ultrathin yttrium iron garnet films. Nat. Mater. 2022, 21, 1352–1356. 10.1038/s41563-022-01369-0. 36138146

[ref42] Peng B.; Li Q.; Liang X.; Song P.; Li J.; He K.; Fu D.; Li Y.; Shen C.; Wang H.; Wang C.; Liu T.; Zhang L.; Lu H.; Wang X.; Zhao J.; Xie J.; Wu M.; Bi L.; Deng L.; Loh K. P. Valley Polarization of Trions and Magnetoresistance in Heterostructures of MoS(2) and Yttrium Iron Garnet. ACS Nano 2017, 11, 12257–12265. 10.1021/acsnano.7b05743. 29182851

[ref43] Wang Y.; Ramaswamy R.; Motapothula M.; Narayanapillai K.; Zhu D.; Yu J.; Venkatesan T.; Yang H. Room-Temperature Giant Charge-to-Spin Conversion at the SrTiO(3)-LaAlO(3) Oxide Interface. Nano Lett. 2017, 17, 7659–7664. 10.1021/acs.nanolett.7b03714. 29112444

[ref44] Soya N.; Katase T.; Ando K. Elastic and Inelastic Spin Transport in SrTiO3-based Magnetic Heterostructure. Adv. Electron. Mater. 2022, 8, 2200232 10.1002/aelm.202200232.

[ref45] Song Q.; Zhang H.; Su T.; Yuan W.; Chen Y.; Xing W.; Shi J.; Sun J.; Han W. Tang Su, Wei Yuan, Yangyang Chen, Wenyu Xing, Jing Shi, Jirong Sun, Wei Han, Observation of inverse Edelstein effect in Rashba-split 2DEG between SrTiO3 and LaAlO3 at room temperature. Sci. Adv. 2017, 3, e1602312 10.1126/sciadv.1602312. 28345050 PMC5357130

[ref46] Tokura Y.; Nagaosa N. Nonreciprocal responses from non-centrosymmetric quantum materials. Nat. Commun. 2018, 9, 3740 10.1038/s41467-018-05759-4. 30218054 PMC6138722

[ref47] Colino J. M.; de Andrés A. Huge magnetoresistance in ultrathin La0.7Ca0.3MnO3 films: The role of superparamagnetic clusters and domain walls. Appl. Phys. Lett. 2005, 87, 142509 10.1063/1.2081139.

[ref48] Liu L.; Moriyama T.; Ralph D. C.; Buhrman R. A. Spin-torque ferromagnetic resonance induced by the spin Hall effect. Phys. Rev. Lett. 2011, 106, 036601 10.1103/PhysRevLett.106.036601. 21405285

[ref49] Rojas-Sánchez J. C.; Cubukcu M.; Jain A.; Vergnaud C.; Portemont C.; Ducruet C.; Barski A.; Marty A.; Vila L.; Attané J. P.; Augendre E.; Desfonds G.; Gambarelli S.; Jaffrès H.; George J. M.; Jamet M. Spin pumping and inverse spin Hall effect in germanium. Phys. Rev. B 2013, 88, 064403 10.1103/PhysRevB.88.064403.

[ref50] Harder M.; Cao Z. X.; Gui Y. S.; Fan X. L.; Hu C. M. Analysis of the line shape of electrically detected ferromagnetic resonance. Phys. Rev. B 2011, 84, 054423 10.1103/PhysRevB.84.054423.

[ref51] Azevedo A.; Vilela-Leão L. H.; Rodríguez-Suárez R. L.; Lacerda Santos A. F.; Rezende S. M. Spin pumping and anisotropic magnetoresistance voltages in magnetic bilayers: Theory and experiment. Phys. Rev. B 2011, 83, 144402 10.1103/PhysRevB.83.144402.

[ref52] Mosendz O.; Pearson J. E.; Fradin F. Y.; Bauer G. E.; Bader S. D.; Hoffmann A. Quantifying spin Hall angles from spin pumping: experiments and theory. Phys. Rev. Lett. 2010, 104, 046601 10.1103/PhysRevLett.104.046601. 20366725

[ref53] Jungfleisch M. B.; Chumak A. V.; Kehlberger A.; Lauer V.; Kim D. H.; Onbasli M. C.; Ross C. A.; Kläui M.; Hillebrands B. Thickness and power dependence of the spin-pumping effect inY3Fe5O12/Pt heterostructures measured by the inverse spin Hall effect. Phys. Rev. B 2015, 91, 134407 10.1103/PhysRevB.91.134407.

[ref54] Nakayama H.; Ando K.; Harii K.; Yoshino T.; Takahashi R.; Kajiwara Y.; Uchida K.; Fujikawa Y.; Saitoh E. Geometry dependence on inverse spin Hall effect induced by spin pumping in Ni81Fe19/Pt films. Phys. Rev. B 2012, 85, 144408 10.1103/PhysRevB.85.144408.

[ref55] Sanchez J. C.; Vila L.; Desfonds G.; Gambarelli S.; Attane J. P.; De Teresa J. M.; Magen C.; Fert A. Spin-to-charge conversion using Rashba coupling at the interface between non-magnetic materials. Nat. Commun. 2013, 4, 2944 10.1038/ncomms3944. 24343336

[ref56] Mendes J. B.; Alves Santos O.; Meireles L. M.; Lacerda R. G.; Vilela-Leao L. H.; Machado F. L.; Rodriguez-Suarez R. L.; Azevedo A.; Rezende S. M. Spin-Current to Charge-Current Conversion and Magnetoresistance in a Hybrid Structure of Graphene and Yttrium Iron Garnet. Phys. Rev. Lett. 2015, 115, 226601 10.1103/PhysRevLett.115.226601. 26650313

[ref57] Zhang W.; Jungfleisch M. B.; Jiang W.; Pearson J. E.; Hoffmann A. Spin pumping and inverse Rashba-Edelstein effect in NiFe/Ag/Bi and NiFe/Ag/Sb. J. Appl. Phys. 2015, 117, 17C727 10.1063/1.4915479.

[ref58] Sousa F.; Tatara G.; Ferreira A. Skew-scattering-induced giant antidamping spin-orbit torques: Collinear and out-of-plane Edelstein effects at two-dimensional material/ferromagnet interfaces. Phys. Rev. Res. 2020, 2, 043401 10.1103/PhysRevResearch.2.043401.

[ref59] Borge J.; Gorini C.; Vignale G.; Raimondi R. Spin Hall and Edelstein effects in metallic films: From two to three dimensions. Phys. Rev. B 2014, 89, 245443 10.1103/PhysRevB.89.245443.

[ref60] Maleki Sheikhabadi A.; Raimondi R.; Shen K. The Edelstein Effect in the Presence of Impurity Spin-Orbit Scattering. Acta Phys. Polym., A 2017, 132, 135–139. 10.12693/APhysPolA.132.135.

[ref61] El Hamdi A.; Chauleau J.-Y.; Boselli M.; Thibault C.; Gorini C.; Smogunov A.; Barreteau C.; Gariglio S.; Triscone J.-M.; Viret M. Observation of the orbital inverse Rashba–Edelstein effect. Nat. Phys. 2023, 19, 1855–1860. 10.1038/s41567-023-02121-4.

[ref62] Jin M. J.; Moon S. Y.; Park J.; Modepalli V.; Jo J.; Kim S. I.; Koo H. C.; Min B. C.; Lee H. W.; Baek S. H.; Yoo J. W. Nonlocal Spin Diffusion Driven by Giant Spin Hall Effect at Oxide Heterointerfaces. Nano Lett. 2017, 17, 36–43. 10.1021/acs.nanolett.6b03050. 27935722

