# Supplementary material for: Interfacial Spin–Orbit-Coupling-Induced Strong Spin-to-Charge Conversion at an All-Oxide Ferromagnetic/Quasi-Two-Dimensional Electron Gas Interface
Source: ACS Appl Mater Interfaces. 2025 Mar 13;17(12):19026–32. doi: 10.1021/acsami.4c20213 (PMC11955945; doi:10.1021/acsami.4c20213)
Supplement: Supplementary file 1 — am4c20213_si_001.pdf [file am4c20213_si_001.pdf]

Supporting information for :

Interfacial spin-orbit-coupling-induced strong spin-to-charge conversion at an all-oxide ferromagnetic /quasi-two-dimensional electron gas interface

Mi-Jin Jin[1,2,*], Guang Yang[2,3], Doo-Seung Um[4], Jacob Linder[5], and Jason W.A. Robinson[2,*]

[1] Center for Multidimensional Carbon Materials (CMCM), Institute for Basic Science (IBS), Ulsan 44919, Republic of Korea
[2] Department of Materials Science & Metallurgy, University of Cambridge, 27 Charles Babbage Road, Cambridge CB3 0FS, United Kingdom
[3] School of Integrated Circuit Science and Engineering, Beihang University, Beijing 100191, China
[4] Department of Electronic Engineering, Jeju National University (JNU), Jeju-do 63243, Korea
[5] Center for Quantum Spintronics, Department of Physics, Norwegian University of Science and Technology, NO-7491 Trondheim, Norway

*Corresponding Author :
Mi-Jin Jin : mijin8276@gmail.com, jinmijin@ibs.re.kr
Jason Robinson : jjr33@cam.ac.uk

Keywords

Spin-Orbit Coupling, Spin Hall effect, Spin-Charge conversion, Oxide Interface, ferromagnetic resonance

1. X-ray diffraction analysis

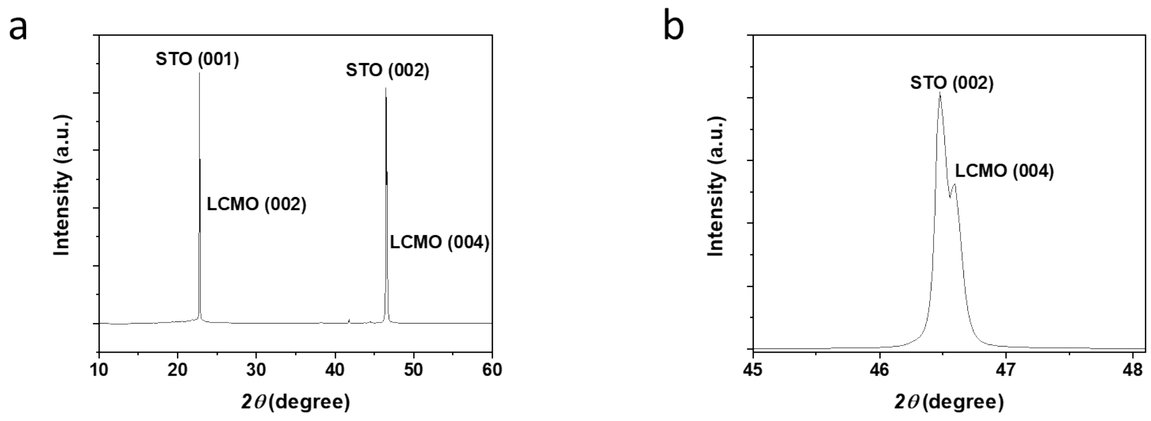

Figure S1. X-ray diffraction analysis by using Grazing incidence X-ray diffraction (GIXRD) technique. LCMO/conducting STO/STO sample. a. 10 ° ~ 60 ° scan angle. b. magnified data at the second peak position.

2. Sample surface

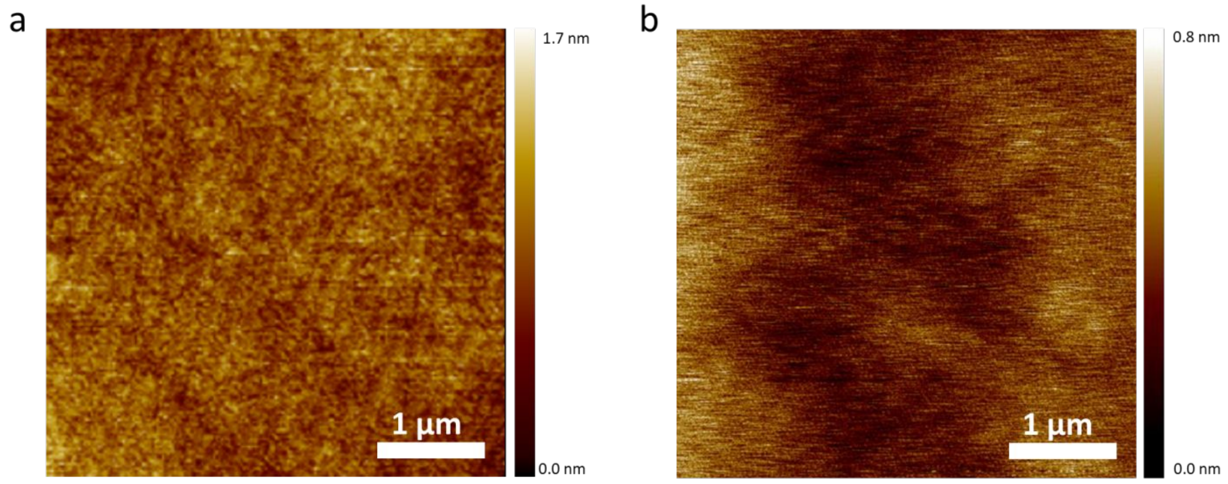

Figure S2. Two different sample surfaces of a. LCMO on TiO2 terminated STO and b. LCMO on conductive STO surface (after plasma treatment)

3. LCMO thin film's temperature dependent sheet resistance

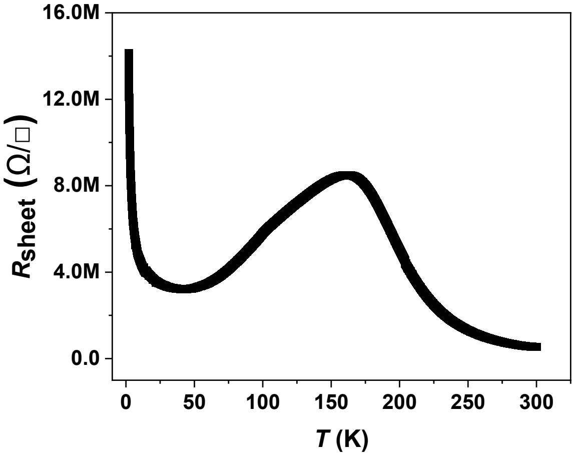

Figure S3. Sheet resistance behavior of LCMO thin film depends on temperature range 300 K – 2 K. I_{source} = 1 μA. The LCMO layer shows mainly semiconductor behavior with slight transition between metal and insulator. Compared with figure 2, this data confirms the quasi-2D conducting interface is well alive and easy to distinguish signals from LCMO or conducting STO interface. At low temperature, the self spin-to-charge conversion cannot (can hardly) be detected because of its high resistance range.

4. Dead layer estimation

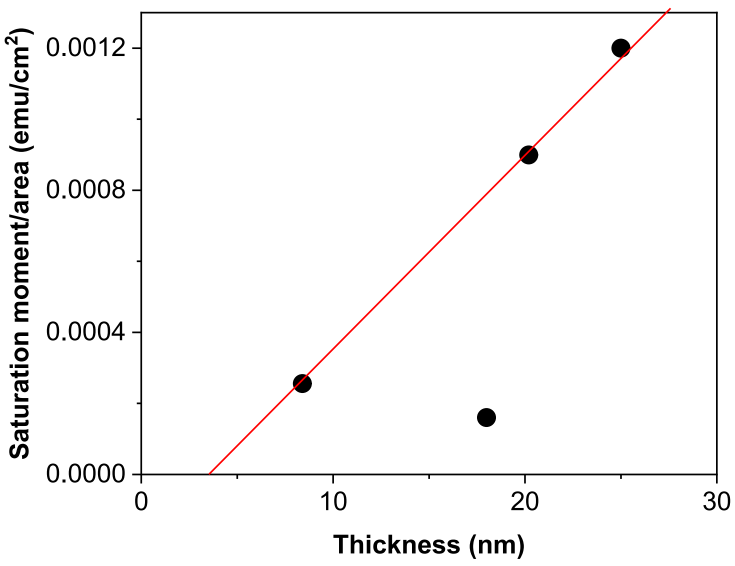

Figure S4. From the saturation magnetic moment/area vs thickness of LCMO, we estimate the magnetic dead layer by using linear fitting relation. It shows magnetic dead layer less than 5 nm.

5. FMR and spin pumping data at 2K, 10K

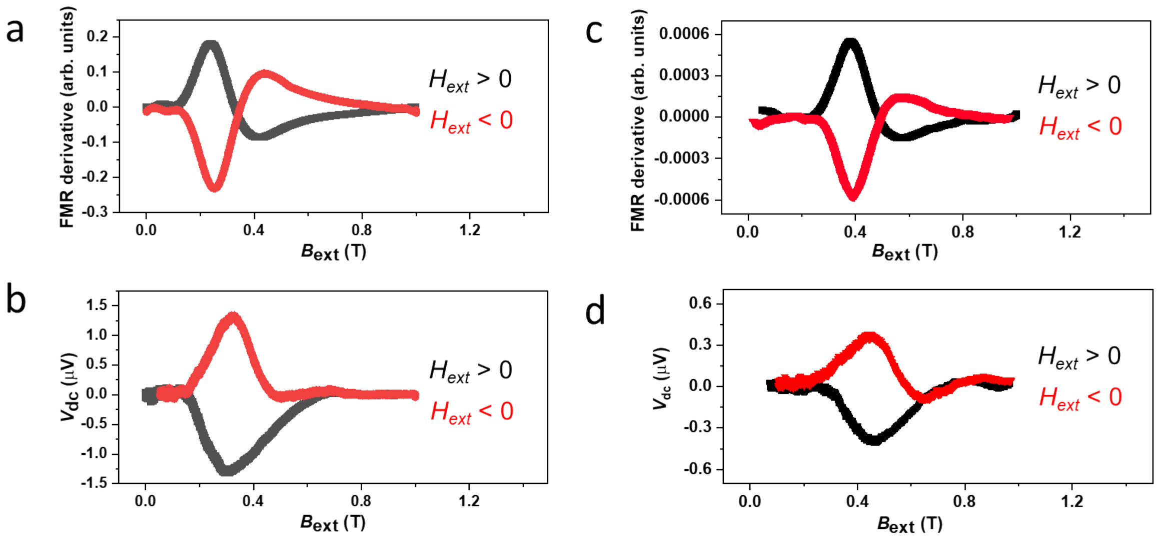

Figure S5. FMR and spin pumping V_{dc} voltage at 2 K (a,b) and 10 K (c,d) temperatures. Both of the FMR (a) and spin pumping signal (b) shows higher sensitivity at 2 K temperature. Also, at 10 K temperature, the asymmetry component seems stronger as shown at figure d (red line)

Temp. (K)	Efficiency (nm)	Material structure	Reference	Comment
300	0.25	NiFe/Ag/Bi	Nat. Commun. 2013, 4, 2944	
300	0.001	$Y_3Fe_5O_{12}$(YIG)/graphene	Phys. Rev. Lett. 2015, 115, 226601	
300	0.11	Py/Ag/Bi	J. Appl. Phys. 2015, 117, 17C727	
300	0.011	Py/Ag/Sb	J. Appl. Phys. 2015, 117, 17C727	
300	0.002	$Y_3Fe_5O_{12}$(YIG)/Bi_2Se_3	Phys.. Rev. Mater. 2021, 5, 024206	
20	6	$La_{0.67}Sr_{0.33}MnO_3/LaAlO_3/SrTiO_3$	Phys. Rev. Res. 2020, 2, 012014 (R)	Variable with temperature
7	6.4	NiFe/LaAlO3/SrTiO3	Nat. Mater. 2016, 15, 1261-1266	
7	20	NiFe/Al/SrTiO3	Nat. Mater. 2019, 18, 1187-1193	Variable with gate voltage
7	50	NiFe/Al/SrTiO3	Nature 2020, 580, 483-486	Variable with gate voltage
15	190	$La_{0.67}Sr_{0.33}MnO_3/La_2Ti_2O_7/SrTiO_3$	Nat. Commun. 2022, 13, 5631	Variable with temperature
5	2.32	$La_{0.7}Ca_{0.3}MnO_3$/conducting $SrTiO_3$	our experiment	

Table S1. Comparison of Spin to charge efficiency originated from IEE
